# Protective Effect of Metalloporphyrins against Cisplatin-Induced Kidney Injury in Mice

**DOI:** 10.1371/journal.pone.0086057

**Published:** 2014-01-14

**Authors:** Hao Pan, Kezhen Shen, Xueping Wang, Hongzhou Meng, Chaojun Wang, Baiye Jin

**Affiliations:** 1 Department of Urology, the First Affiliated Hospital, College of Medicine, Zhejiang University, Hangzhou, Zhejiang, China; 2 Key Laboratory of Combined Multi-Organ Transplantation, Ministry of Public Health, Hangzhou, Zhejiang, China; University of Sao Paulo Medical School, Brazil

## Abstract

Oxidative and nitrative stress is a well-known phenomenon in cisplatin-induced nephrotoxicity. The purpose of this work is to study the role of two metalloporphyrins (FeTMPyP and MnTBAP), water soluble complexes, in cisplatin-induced renal damage and their ability to scavenge peroxynitrite. In cisplatin-induced nephropathy study in mice, renal nitrative stress was evident by the increase in protein nitration. Cisplatin-induced nephrotoxicity was also evident by the histological damage from the loss of the proximal tubular brush border, blebbing of apical membranes, tubular epithelial cell detachment from the basement membrane, or intra-luminal aggregation of cells and proteins and by the increase in blood urea nitrogen and serum creatinine. Cisplatin-induced apoptosis and cell death as shown by Caspase 3 assessments, TUNEL staining and DNA fragmentation Cisplatin-induced nitrative stress, apoptosis and nephrotoxicity were attenuated by both metalloporphyrins. Heme oxygenase (HO-1) also plays a critical role in metalloporphyrin-mediated protection of cisplatin-induced nephrotoxicity. It is evident that nitrative stress plays a critical role in cisplatin-induced nephrotoxicity in mice. Our data suggest that peroxynitrite is involved, at least in part, in cisplatin-induced nephrotoxicity and protein nitration and cisplatin-induced nephrotoxicity can be prevented with the use of metalloporphyrins.

## Introduction

Cisplatin (cis-diammine-dichloro-platinum) is an inorganic platinum compound with broad-spectrum anti-neoplastic activity against different types of human tumors, particularly solid tumors. However, severe side effects of cisplatin such as nephrotoxicity, neurotoxicity, ototoxicity, greatly hamper its chemotherapeutic efficacy [1]. The exact mechanisms of the side effects induced by cisplatin are not clearly understood. It is known that oxidative stress, i.e., the production of reactive oxygen species (ROS) is implicated in the progression of certain side effects [2]. Nitric oxide plays important role in cisplatin induced nephrotoxicity [3] as well as other ROS species such as superoxide anion and hydrogen peroxide are involved [4], [5]. If effected cells in kidney produce both nitric oxide and superoxide with cisplatin, then peroxynitrite must exist [6].

Metalloporphyrins are more efficient among several classes of direct-reacting peroxynitrite scavenger compounds. In 1996, Stern et al. reported that Fe(III) tetra-(N-methyl-4′-pyridyl)-porphyrin (FeTMPyP) catalytically decomposed peroxynitrite almost exclusively to nitrate and proposed that FeTMPyP could function as a “peroxynitrite isomerase” and Iron porphyrins can indeed reduce peroxynitrite in a catalytic manner [30]. Also in 1996, Szabo et al. reported that Mn(III) tetrakis-(4-benzoic acid)-porphyrin (MnTBAP) inhibited peroxynitrite-mediated oxidation and prevented the suppression of mitochondrial respiration in cells exposed to peroxynitrite or NO [7]. Several manganese and iron porphyrins have been reported to prevent NO-dependent oxidative tissue injury in animal models [8], [9].

Previously, various agents have shown protective effect in cisplatin induced nephrotoxicity in mice and rats. Importance of HO-1(Heme oxygenase-1) expression in cisplatin-induced renal injury has been demonstrated using transgenic mice deficient in HO-1 [10]. Transcriptional regulator of HO-1, NRF2 also plays role in cisplatin induced nephropathy as expected [11], [12], [13]. HO-1 and autophagy has been implicated in protective effect of luteolin and berberine in cisplatin induced kidney injury [14], [15]. Cannabinoids and its receptor have protective effect in cisplatin induced kidney failure where inflammation was found to be key regulator of toxicity and cell death [16], [17], [18]. Sulforaphane, a natural constituent of broccoli, prevents cell death and inflammation in cisplatin induced nephropathy in rats [19]. NADPH oxidase is one of the contributor of cisplatin induced superoxide generation and administration of apocynin in drinking water protects against cisplatin toxicity in rats [20]. The same group also reported the protective effect of FeTPPS, an iron based peroxynitrite scavenger, in a rat model of cisplatin toxicity [21]. Recently, mitochondrial antioxidants and downstream PARP inhibitors demonstrate protective effect against cisplatin toxicity in mice [22], [23].

Here, we demonstrated the protection of kidney from cisplatin induced nitrative damage and cell death by two metalloporphyrins. We used a well-established mouse model of cisplatin-induced nephropathy. The results indicated that peroxynitrite induced apoptosis and cell death was the major cause of cisplatin induced kidney injury. The mechanism of protection was also mediated through HO-1. Our results may have important relevance for the prevention of the cisplatin induced nephrotoxicity.

## Materials and Methods

### Ethics Statement

This study was performed in strict accordance with the recommendations of the Guide for the Care and Use of Laboratory Animals of the National Institutes of Health. The protocol was approved by the Committee on the Ethics of Animal Experiments of First Affiliated Hospital, College of Medicine, Zhejiang University (Permit Number: 09-028). The mice were sacrifice by cervical dislocation under anesthesia. All efforts were made to minimize suffering.

### Experiments with Mice

The mouse strain used in the present study was C57BL/6. Eight-week-old male animals with weights of 18–22 g were used in all experiments. Generally, the experimental groups were composed of at least six mice. Animals from the same experimental group were kept in the same cage under constant temperature (22°C) and humidity with a 12-h light/dark cycle and had access to food and water *ad libitum* throughout the study. All procedures, care and handling of animals were approved by the Ethics Committee of the Zhejiang University at the Chinese Academy of Sciences.

Mice were sacrificed 3 days (72 hours) after a single injection of cisplatin (cis-diammineplatinum (II) dichloride, Sigma) at dose 20 mg/kg i.p. Two metalloporphyrins MnTBAP (Mn(III)tetrakis(4-benzoic acid) porphyrin chloride) and FeTMPyP (Fe(III)tetrakis (1-methyl-4-pyridyl) porphyrin pentachlorideporphyrin pentachloride) were purchased from Cayman Chemical (NeoBioscience Technology, Shenzhen, China) The metalloporphyrins MnTBAP and FeTMPyP were dissolved in saline and administered at 10 mg/kg (or as described in text), i.p, daily, starting 3 h before the cisplatin administration. Two separate cohort studies were carried out with administration of metalloporphyrins 12 hour post cisplatin administration and 24 h/48 h before cisplatin administration.

For each set of experiments, six mice were taken for each group. The experiments were repeated two more times.

### Serum Measurements

On the day of the sacrifice, blood was immediately collected and serum levels of blood urea nitrogen (BUN) and creatinine were measured using a blood chemistry analyzer.

### Histological Evaluation of Kidney

Kidneys were fixed with 10% formalin for 24 hours. Kidneys were sectioned and stained with periodic acid–Schiff (PAS) reagents for histological examination. Tubular damage in PAS-stained sections was examined under the microscope and scored based on the percentage of cortical tubules showing epithelial necrosis: 0-normal; 1<10%; 2–10–25%; 3–26–75%; 4>75%. Tubular necrosis was defined as the loss of the proximal tubular brush border, blebbing of apical membranes, tubular epithelial cell detachment from the basement membrane, or intra-luminal aggregation of cells and proteins. The morphometric examinations were performed in a blinded manner.

For cleaved caspase 3 staining (early marker of apoptosis) slides were deparaffinized and hydrated in descending gradations of ethanol, followed by antigen retrieval. Sections were incubated in 0.3% H2O2 in PBS to block endogenous peroxidase activity and incubated with anti-cleaved caspase 3 (Cell Signaling Technology) antibodies overnight in a moist chamber. Biotinylated secondary antibodies and ABC reagent were added as per the kit's instructions (Vector Laboratories, USA). Color development was induced by incubation with a DAB kit (Vector Laboratories) for 5 min.

Apoptosis was assessed by TUNEL, and the number of apoptotic cells, as defined by nuclear fragmentation was counted. Apoptosis was detected in the kidneys by TUNEL assay according to manufacturer’s instructions (Roche China Ltd., Shanghai). The histological examinations were performed in a blinded manner.

### Quantitative Measurement of Protein Nitro-tyrosine in Kidney

Nitrotyrosine content was evaluated by ELISA. Briefly, an identical amount of protein from cell lysates was applied to a Maxisorp ELISA plate together with nitrated BSA (Bovine serum albumin) standard and allowed to bind overnight at 4°C. After blocking with 2% BSA in PBS(phosphate buffered saline), wells were incubated at 37°C for 2 h with a mouse monoclonal antibody anti-nitrotyrosine (Upstate Biotechnology, Lake Placid) and then for 1 h at 37°C with a peroxidase conjugated goat anti-mouse IgG secondary antibody. After washing, peroxidase reaction product was generated using 3, 30, 5 50 -tetramethylbenzidine (TMB) peroxidase substrate.

### Quantitative Measurement of Apoptosis in Kidney

Caspase-3/7 activity of the lysate was measured using Apo- One Homogenous caspase-3/7 Assay Kit (Promega Corp., Madison, WI, USA). An aliquot of caspase reagent was added to each well, mixed on a plate shaker for 2 h at 37°C in dark and the fluorescence was measured.

### Quantitative Measurement of DNA Fragmentation in Kidney

The DNA fragmentation assay is measured in the cytoplasmic fraction of tissue extracts using a commercially available kit (Roche China Ltd., Shanghai) according to manufacturer’s instructions.

### Real-time PCR of HO-1

Isolation and Real-time PCR were carried out as described earlier [18]. The primer sets for HO-1 and actin were purchased from Qiagen (Pudong, Shanghai, China).

### Statistical Analysis

All data were presented as the means ± SEMs. Two-sample comparisons were performed using Student’s t-tests.

## Results

### Metalloporphyrins Attenuates the Cisplatin-induced Renal Dysfunction in Mice

**Figure 1:**
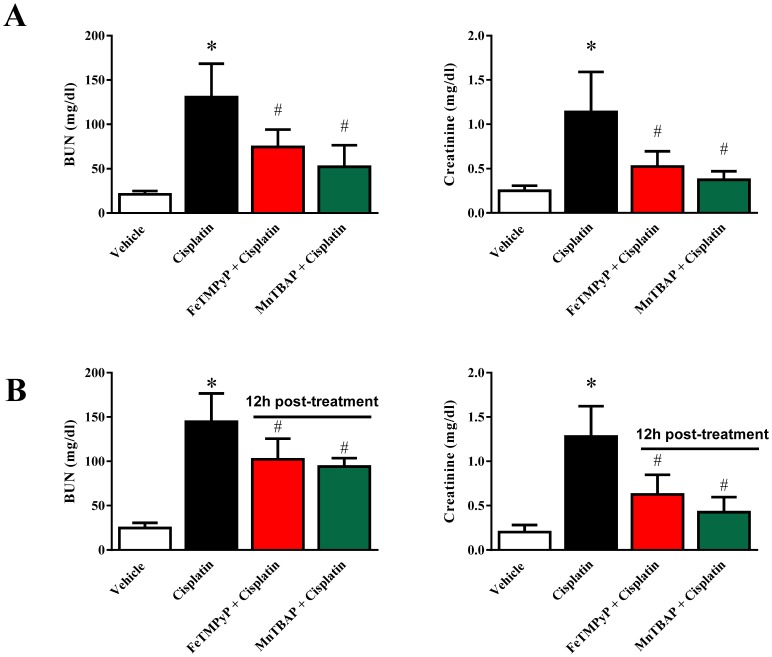
Effects of metalloporphyrins on Cisplatin induced renal dysfunction in mice. Cisplatin-induced renal dysfunction was measured by the levels of BUN and creatinine. A. BUN and Creatinine levels were measured at 72 h after cisplatin administration. Cisplatin administration resulted in severe kidney injuries which were attenuated by FeTMPyP and MnTBAP treatments. B. BUN and Creatinine levels were measured at 72 h after cisplatin administration. FeTMPyP and MnTBAP were administered 12 hour after cisplatin treatment. Results are mean ± S.E.M. n = 4–5/group. *p<0.05 versus vehicle; and ^#^p<0.05 versus cisplatin.

To investigate the effect of metalloporphyrins (FeTMPyP and MnTBAP) on cisplatin-induced renal dysfunction, levels of BUN (Blood Urea Nitrogen) and creatinine were measured at 72 h after cisplatin administration in the mice serum. Cisplatin administration resulted in severe kidney injury (increase of 6.2 fold BUN and 4.6 fold creatinine) as shown in Figure 1A, which was attenuated by FeTMPyP and MnTBAP treatment (n = 4–5/each group; p<0.05). The doses of metalloporphyrins were optimized based on earlier dose dependent studies (Figure S1). In addition, both scavengers were administered 12 hour post cisplatin injection and provided partial protection (Figure 1B). When FeTMPyP and MnTBAP were administered 24 h or 48 h before cisplatin injection as a pre-schedule, there were no statistically significant difference in BUN and Creatine levels ((Figure S2).

### Metalloporphyrins Attenuates Cisplatin-induced Kidney Tubular Damage in Mice

**Figure 2:**
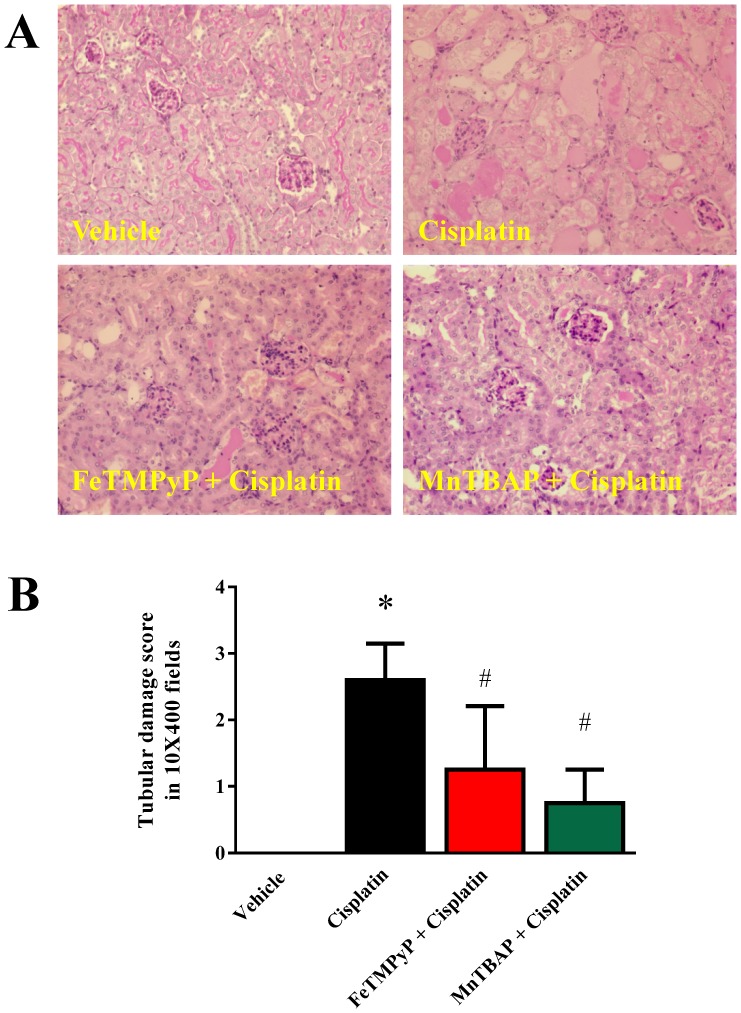
Effects of metalloporphyrins on Cisplatin induced kidney tubular damage in mice. Histological examination (Panel A) and quantification (Panel B) revealed necrosis, protein cast, vacuolation, and desquamation of epithelial cells in the renal tubules of the cisplatin-treated group. Cisplatin administration resulted in severe tubular damage. Cisplatin induced damages were attenuated by FeTMPyP and MnTBAP treatments (n = 4–5/each group; p<0.01). Results are mean ± S.E.M. n = 4–5/group. *p<0.05 versus vehicle; and ^#^p<0.05 versus cisplatin.

Histological examination revealed necrosis, protein cast, vacuolation, and desquamation of epithelial cells in the renal tubules of the cisplatin-treated group. Cisplatin administration resulted in severe tubular damage with average score 2.6 as shown in Figure 2, which was attenuated by FeTMPyP (52% decrease) or MnTBAP (72% decrease) treatment.

### Metalloporphyrins Attenuates Cisplatin-induced Nitrative Stress in Mice

**Figure 3:**
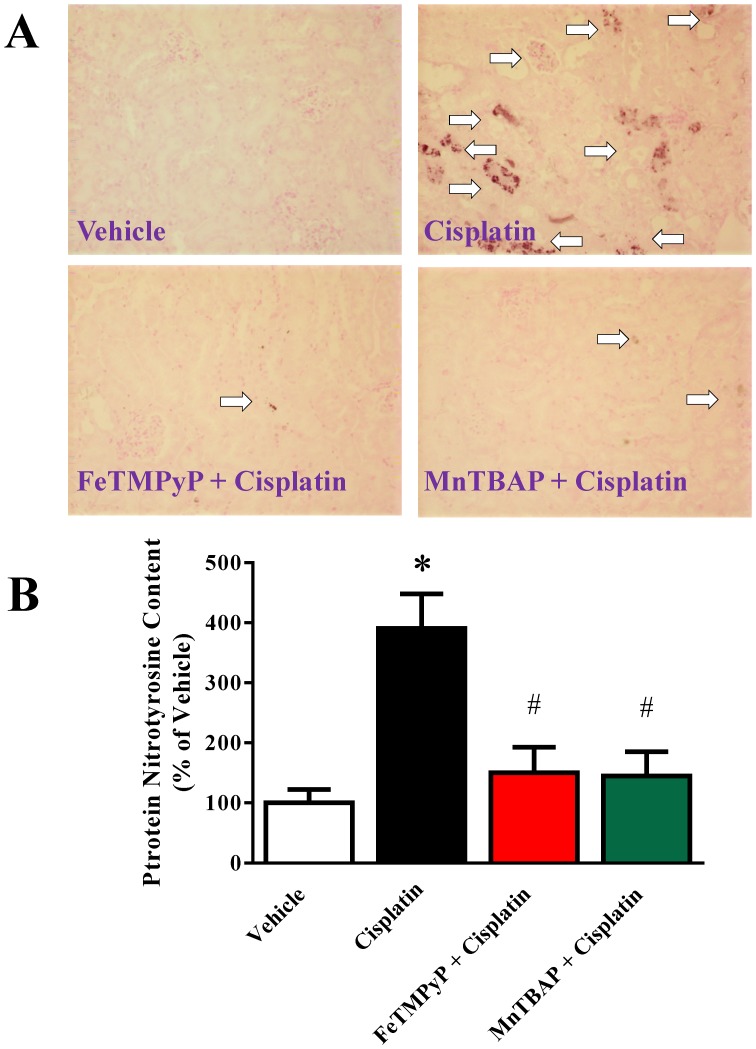
Effects of metalloporphyrins on Cisplatin induced nitrative stress in mice. Panel A: Histological examination revealed significant protein nitration in the renal tubules of the cisplatin-treated group. FeTMPyP and MnTBAP treatments lead to reduced protein nitration similar to vehicle level (n = 4–5/each group; p<0.01). Panel B: Quantitative measurement of protein nitration also demonstrated 3.9 fold increased by cisplatin and the protein nitration is decreased to 1.5 and 1.4 fold for FeTMPyP and MnTBAP treated groups respectively. Results are mean ± S.E.M. n = 4–5/group. *p<0.05 versus vehicle; and ^#^p<0.05 versus cisplatin.

Histological examination revealed significant protein nitration in the renal tubules of the cisplatin-treated group. FeTMPyP and MnTBAP treatment reduced protein nitration similar to vehicle level (Figure 3). Quantitative measurement of protein nitration also demonstrated 3.9 fold increased by cisplatin and the protein nitration was decreased to 1.5 and 1.4 fold for FeTMPyP and MnTBAP treated groups respectively.

### Metalloporphyrins Attenuates Cisplatin-induced Early Apoptosis Marker Caspase 3 in Mice

**Figure 4:**
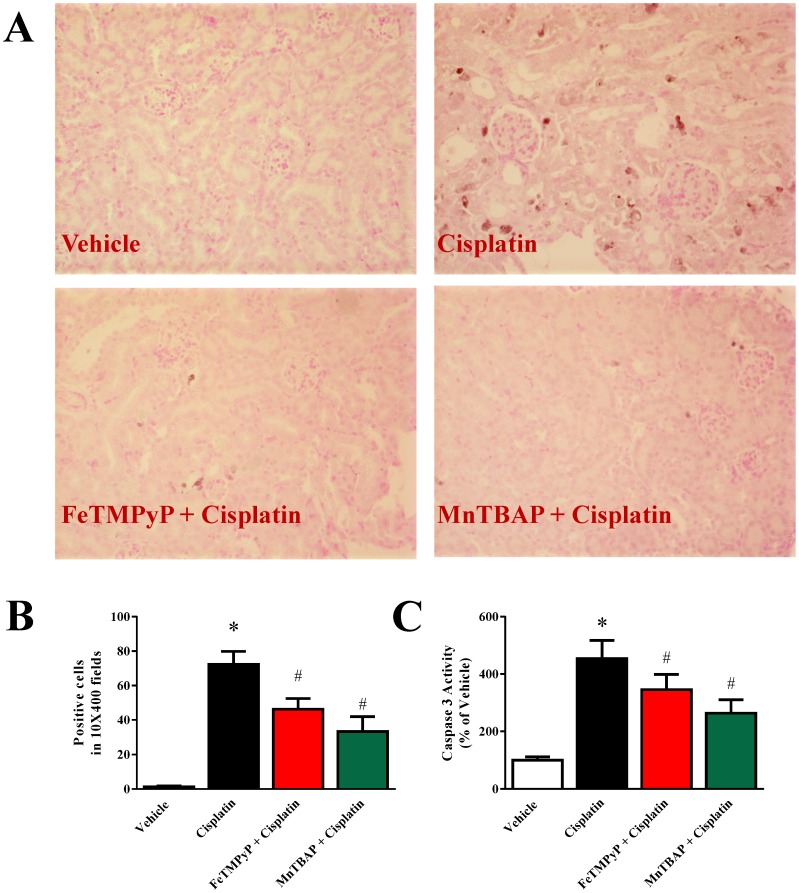
Effects of metalloporphyrins on Cisplatin induced early apoptosis marker Caspase 3. Histological examination (Panel A) demonstrated cisplatin induced cleaved caspase 3 staining in kidney and the caspase 3 staining were significantly attenuated with pretreatments of FeTMPyP or MnTBAP. Panel B: caspase 3 activity were measured and similar trend was observed. Results are mean ± S.E.M. n = 4–5/group. *p<0.05 versus vehicle; and ^#^p<0.05 versus cisplatin.

Activation of caspase-3 required proteolytic processing of the inactive zymogen into p18 and p12 subunits and used as marker for early apoptosis. Here, antibody to cleaved caspase 3 was used for histological analyses. As shown in figure 4, cisplatin induced cleaved caspase 3 staining in mice kidney and the staining is significantly attenuated when mice were pretreated with FeTMPyP or MnTBAP. In addition to that, caspase 3 activity were also measured. Cisplatin induced caspase 3 activity 4.5 fold compared to vehicle and the activity was reduced by 24% and 42% with FeTMPyP and MnTBAP treatments respectively.

### Metalloporphyrins Attenuates the Cisplatin-induced Tubular Apoptosis and DNA Fragmentation

**Figure 5:**
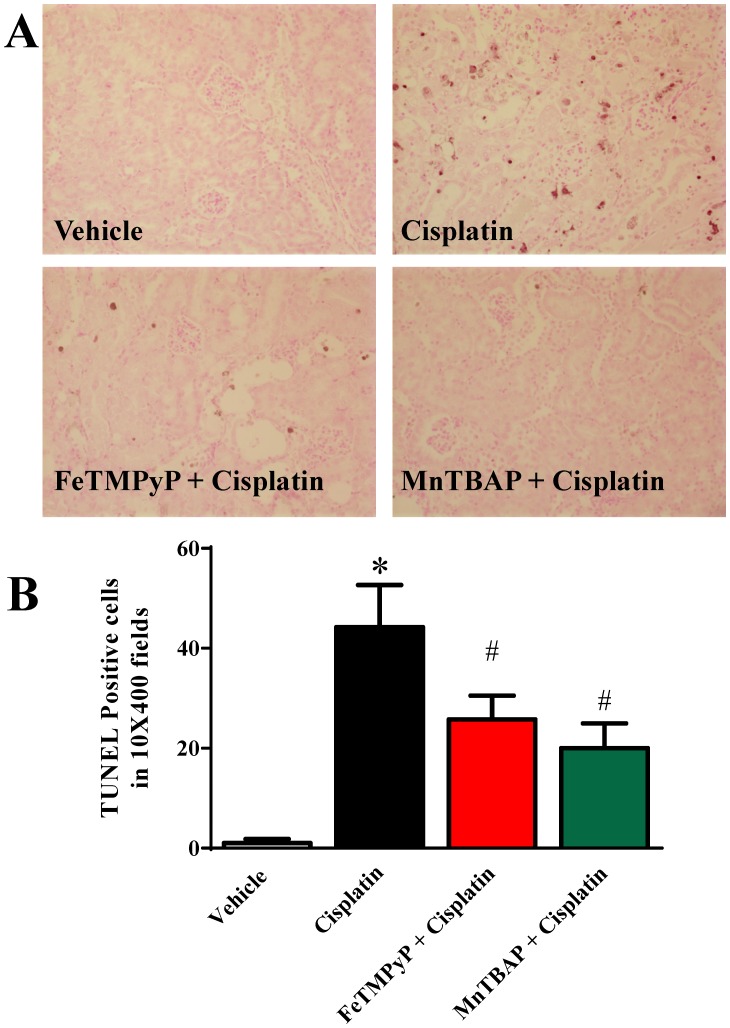
Effects of metalloporphyrins on Cisplatin induced tubular apoptosis. TUNEL-positive apoptotic cell numbers were also increased in cisplatin-treated mice and attenuated by FeTMPyP or MnTBAP treatments.

**Figure 6:**
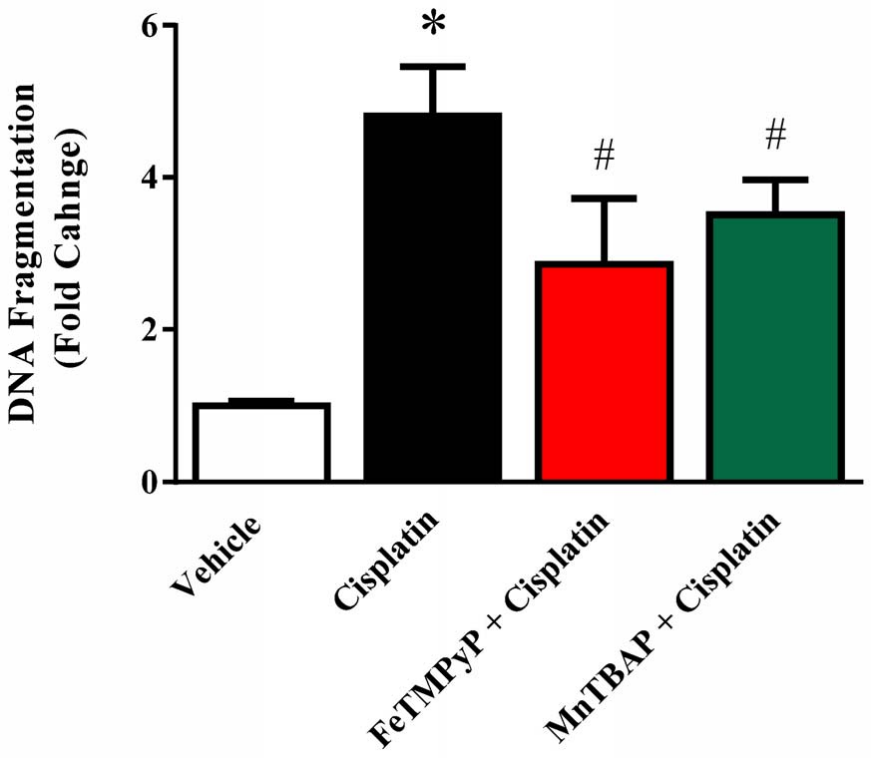
Effects of metalloporphyrins on Cisplatin induced DNA fragmentation. Cisplatin induced significant DNA fragmentation and the effect was attenuated by FeTMpyP or MnTBAP treatments. Results are mean ± S.E.M. n = 4/group. *p<0.05 versus vehicle; and ^#^p<0.05 versus cisplatin.

Apoptosis of renal tubular epithelial cells was evaluated by TUNEL; method for detecting DNA nicks that result from apoptotic signaling cascades. TUNEL-positive apoptotic cell numbers were also increased in cisplatin-treated mice and attenuated by FeTMPyP or MnTBAP treatment (Figure 5). As shown in figure 6, DNA fragmentation in kidney homogenates were markedly increased to 4.8 fold after cisplatin administration and significantly attenuated by FeTMPyP (2.8 fold) or MnTBAP (3.5 fold) treatment (n = 4; p<0.05).

### Metalloporphyrins Attenuates the Cisplatin-induced Heme Oxygenase 1 Gene

**Figure 7:**
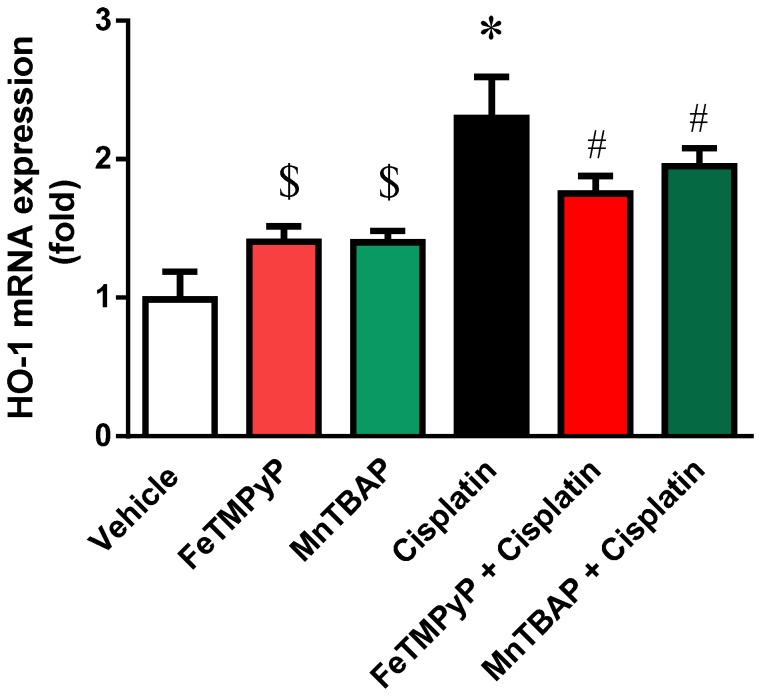
Effects of metalloporphyrins on Cisplatin induced HO-1 mRNA. Metallophorphyrins induced HO-1 mRNA by itself ($ p<0.05 vs vehicle). Cisplatin induced significant HO-1 mRNA level and the induction was attenuated by FeTMpyP or MnTBAP treatments. Results are mean ± S.E.M. n = 4/group. *p<0.05 versus vehicle; and ^#^p<0.05 versus cisplatin.

Both metalloporphyrins induced HO-1 gene when administered alone in mice. However, the level of HO-1 induction is higher in cisplatin-treated mice and attenuated by FeTMPyP or MnTBAP treatment (Figure 7).

## Discussion

In this present study, we investigated whether metalloporphyrins could protect against cisplatin-induced nephrotoxicity using a preclinical mouse model. We demonstrated that two hours pretreatments with metalloporphyrins FeTMPyP and MnTBAP attenuated cisplatin-induced kidney injury by reducing cellular oxidative/nitrative stress, caspase 3 activity, DNA fragmentation and apoptosis. These metalloporphyrins also modulated HO-1 mRNA level contributing to protection against cisplatin induced oxidative injury. In addition to that, administration of FeTMPyP and MnTBAP 12 h after cisplatin injection leads to partial protection. Acute kidney injury is increasingly prevalent in developing as well as developed countries and is associated with severe morbidity and mortality [24]. Acute kidney injury is also associated with cancer patients undergoing cisplatin chemotherapy. Cisplatin is an anti-neoplastic drug used in the treatment of many solid-organ cancers, including those of the head, neck, testis, ovary, lung and breast. While toxicities include ototoxicity, gastrotoxicity, and allergic reactions, the main side effect of cisplatin is dose dependent nephrotoxicity [25], [26], [27]. Despite intense efforts over past three decades to find less toxic but equally effective alternatives, cisplatin continues to be widely prescribed. Searching of the NIH ClinicalTrials.gov database returned 742 active treatment trials involving cisplatin as an indication of its ongoing wide clinical use. In the present study we assessed two metalloporphyrins FeTMPyP and MnTBAP on cisplatin induced renal oxidative stress, cell death and subsequent kidney dysfunction in mice. Both of these porphyrins attenuated cisplatin induced oxidative injury, cell death and kidney dysfunction.

Cisplatin is considered as a potent generator of reactive oxygen/nitrogen species that causes oxidative stress injury and triggers cell death [28]. Our results demonstrated that cisplatin treatment induced protein nitration in mice kidneys, which is in agreement with previous findings [12], [15], [21]. Nitric oxide is well known regulator of physiological processes [29]. However, the overproduction of nitric oxide and enhanced superoxide generation, result in the formation of short lived but hyperactive species peroxynitrite and subsequently nitration of protein tyrosine residues. Peroxynitrite anions are formed from the diffusion-controlled reaction between nitric oxide and superoxide. Peroxynitrite anions are strong oxidants and nitrating species that promote oxidative damage by a variety of mechanisms [30]. One of its target, protein nitration leads to cell damage and organ dysfunction [29]. Thus, the scavengers of peroxynitrite represent potential therapeutic approach to oxidative tissue injury [31]. In this study, both metalloporphyrins FeTMPyP and MnTBAP completely ameliorated nitrative stress, suggesting strong antioxidant and nephroprotective activity.

Manganese-porphyrins(MnTBAP) have been demonstrated to have protective effects against peroxynitrite-mediated cytotoxicity in vitro [32]. However, its protective effects in vivo are probably due to multiple antioxidant effect. There are several mechanisms by which MnTBAP could be protective against cisplatin induced nephrotoxicity: First, by its SOD mimetic effect [7], second, by preserving SOD activity [33] and third by directly scavenging peroxynitrite [34], [35]. In all mechanisms, MnTBAP indirectly (SOD activity leads to decreased superoxide) or directly diminished peroxynitrite levels [36]. In contrast, FeTMPyP catalyzes the decomposition of peroxynitrite to nitrate *in vivo* [37], [38], [39]. In addition, it has been demonstrated the important role of iron in cisplatin-induced nephrotoxicity [40]. We attempted here to quantify protein nitrotyrosine formation to confirm that MnTBAP and FeTMPyP was effective in its role as a peroxynitrite scavenger and we demonstrated that protein nitration level were significantly reduced from cisplatin treated group by both metalloporphyrins. Thus, our results supported the hypothesis that MnTBAP and FeTMPyP indeed reduced the level of peroxynitrite in cisplatin induced *in vivo* model of kidney injury. Accumulating evidence indicates that peroxynitrite, formed from the diffusion-controlled reaction between nitric oxide and the superoxide radical represents a major oxidant and nitrating species triggering significant renal damage in this microenvironment [41]. Peroxynitrite is a powerful oxidant which is highly reactive towards biological molecules including protein and non-protein sulfhydryl, DNA, and membrane phospholipids [42], [43], [44]. Peroxynitrite is also stable enough to cross several cell diameters to reach targets before becoming protonated and decomposing [45].

ROS generation in cisplatin induced kidney injury are contributed to many sources such as NADPH oxidases [20], NOX2, NOX4 [18] and mitochondrial electron transport chain [22]. Nitric oxide is mainly generated from iNOS [18]. Despite the important role of each ROS producing sources from NADPH oxidase to mitochondria, it is difficult to conceive the sole contribution of one source over the other. Our results suggest that the cisplatin induced ROS generation is a complex process involving multiple enzymes. Recent time course studies in cisplatin induced nephrotoxicity demonstrate the possible role of mitochondria in early event compared to other non-mitochondrial enzymatic ROS generation [23].

The effect for peroxynitrite modification in the biological molecules is apoptotic cell death. We have observed significant histopathological staining of cleaved caspase 3, a powerful known marker of early apoptosis, in the tubular cells of kidney treated with cisplatin. Similar data was observed with TUNEL staining. One of the key steps in apoptotic pathway is participation of mitochondria and cisplatin-induced peroxynitrite formation leads to mitochondrial damages. It involves the potential nitration of cytochrome c during peroxynitrite interactions with mitochondria. Peroxynitrite induces cytochrome c nitration at critical tyrosine-67 [46] and nitrated cytochrome c may be found in the cytosol of cells undergoing apoptosis [47]. Other mitochondrial proteins are known to be target of peroxynitrite such as aconitase, manganese superoxide dismutase, creatine kinase [48], [49], [50]. Mitochondria is the involved in intrinsic pathway of apoptotic cell death mediated by cisplatin. In the present study, both metalloporphrins reduced cisplatin induced peroxynitrite levels and associated damage to mitochondria in kidney.

One of interesting observation is the induction of HO-1 in mice kidney when administered with metalloporphyrins as observed earlier [51]. The stress protein heme oxygenase-1 (HO-1) is a major stress inducible protein in mammalian cells [52] and its transcriptional up regulation responds to a broad spectrum of chemical and physical stress, and represents a general cellular response to oxidative stress [53] HO-1 is generally considered a cytoprotective enzyme, and can confer protection in various models of experimental tissue injury [54] Recent cisplatin studies have now shown that the HO-1 can also modulate the regulation of autophagy [15]. Induction of HO-1 by metalloporphyrins may lead reduced damage by cisplatin as antioxidant defense plays a critical role in the protection as compelling evidence. However, this may not be the only mechanism by which metalloporphyrins protect against cisplatin induced kidney injury. This lead to hypothesis of multilevel protection by metalloporphyrins in cisplatin induced nephrotoxicity. One of key component in multilevel protection is modulation of HO-1 by FeTMPyP and MnTBAP, a common target reported earlier in berberine or epigallocatechin-3-gallate or luteolin mediated protection against cisplatin toxicity [12], [15], [55].

One of our key observations was partial protection of cisplatin induced kidney injury by administration of FeTMPyP and MnTBAP 12 hour after cisplatin injection. This leads to the possibility that peroxynitrite formation might have peaked after 12 hour after cisplatin administration. Indeed, earlier time course studies demonstrated that increase in protein nitration (marker for peroxynitrite) doubled after 24 hours [23]. Similar post treatment protection were not reported earlier particularly in Rosiglitazone mediated protection [56], [57], [58], [59]. Rosiglitazone is PPARγ agonist that has been reported to have anti-inflammatory action by modulating transcription factors NFKB, NF-AT,Sp1 and AP-1 [60]. We have not observed any statistically significant difference when FeTMPyP or MnTBAP were administered 24 h or 48 h before cisplatin administration.The difference with rosiglitazone in conferring protection against cisplatin might attribute to multiple factors such as retention time of the drug, different transcriptional response and involvement of signaling cascade pathways.

The role of inflammation in cisplatin nephrotoxicity is crucial and is another component of our hypothesis. The protective effects of sulforaphane, cannabidiol, CB(cannabinoid receptor)-2 agonists and rosiglitazone against cisplatin nephrotoxicity are mediated by suppressing the inflammatory response involving TNF alpha, IL-10 and other cytokines [17], [18], [19], [56]. Inflammatory response is observed in response to cell death mediated by cisplatin induced oxidative tissue injury. Apocyanin was observed to reduce the damaging effect of cisplatin by inhibiting ROS generation (NADPH oxidase) [20]. Similarly, FeTMPyP and MnTBAP reduced oxidative tissue injury and associated inflammatory response.

**Figure 8:**
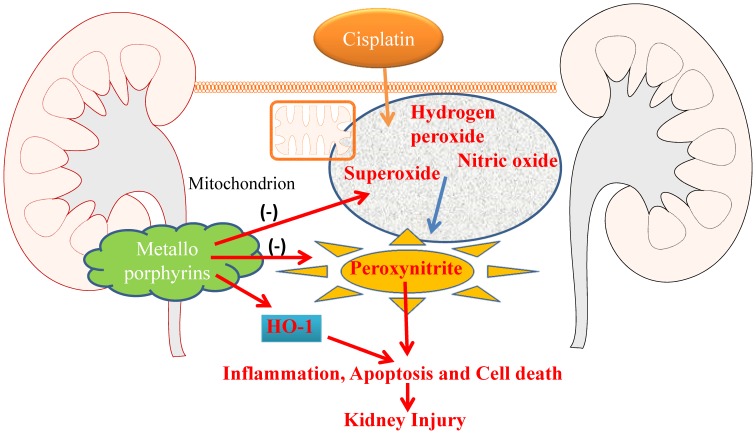
Schematic diagram of protection mechanism of metalloporphyrins in Cisplatin induced kidney injury. Metalloporphyrins induce HO-1 resulting in antioxidant defense and autophagy during cisplatin induced kidney injury. Metalloporphyrins also neutralize superoxide or scavenge peroxynitrite generated during cisplatin exposure. All combitorial effects leads to reduced inflammation and cell death, thus protecting against cisplatin induced kidney injury.

In summary, the putative mechanism of metalloporphyrins is at multilevel protection as follows: (1) inducing antioxidant defense mechanism through HO-1 (2) directly neutralizing superoxide as SOD-mimetic and (2) by scavenging the most damaging radical peroxynitrite (Figure 8). These combined multi-level mechanisms leads to limited cell death and inflammation by FeTMPyP and MnTBAP in cisplatin induced kidney injury.

Metalloporphyrins as peroxynitrite decomposition scavengers have been shown to be effective in a variety of disease models [61], [62], [63], [64], [65], and are promising candidates for clinical trials to prevent oxidative tissue injury and inflammatory response syndrome, especially given the apparently relative broad window of opportunity for their use. Although FeTMPyP and MnTBAP afforded protection in our model, it warrants further investigation for use as therapeutic potential.

## Supporting Information

Figure S1 **Dose Dependent effect of Metalloporphyrins on Cisplatin-Induced Renal Dysfunction in Mice.** Cisplatin-induced significant renal dysfunction measured by the levels of BUN and creatinine. BUN and Creatinine were measured in serum from mice at 72 h after cisplatin administration. Cisplatin administration resulted in severe kidney injury which was attenuated by FeTMPyP and MnTBAP treatment in a dose dependent manner. Results are mean ± S.E.M. n = 4–5/group. *p<0.05 versus vehicle; and ^#^p<0.05 versus cisplatin.
(TIF)

Figure S2 **Effect of Metalloporphyrins pretreatments on Cisplatin-Induced Renal Dysfunction in Mice.** Cisplatin-induced significant renal dysfunction measured by the levels of BUN and Creatinine. BUN and Creatinine were measured in serum from mice at 72 h after cisplatin administration. Cisplatin administration resulted in severe kidney injury which was not attenuated by FeTMPyP and MnTBAP treatment when administered at 24 h or 48 h before cisplatin administration. Results are mean ± S.E.M. n = 4/group. *p<0.05 versus vehicle; and ^#^p<0.05 versus cisplatin.
(TIF)

## References

[1] Fram RJ (1992) Cisplatin and platinum analogues: recent advances. Curr Opin Oncol 4: 1073–1079. 1457521 10.1097/00001622-199212000-00012

[2] Dillioglugil MO, Maral Kir H, Gulkac MD, Ozon Kanli A, Ozdogan HK, et al (2005) Protective effects of increasing vitamin E and a doses on cisplatin-induced oxidative damage to kidney tissue in rats. Urol Int 75: 340–344. 16327303 10.1159/000089171

[3] Srivastava RC, Farookh A, Ahmad N, Misra M, Hasan SK, et al (1996) Evidence for the involvement of nitric oxide in cisplatin-induced toxicity in rats. Biometals 9: 139–142. 8744896 10.1007/BF00144618

[4] Davis CA, Nick HS, Agarwal A (2001) Manganese superoxide dismutase attenuates Cisplatin-induced renal injury: importance of superoxide. J Am Soc Nephrol 12: 2683–2690. 11729237 10.1681/ASN.V12122683

[5] Kadikoylu G, Bolaman Z, Demir S, Balkaya M, Akalin N, et al (2004) The effects of desferrioxamine on cisplatin-induced lipid peroxidation and the activities of antioxidant enzymes in rat kidneys. Hum Exp Toxicol 23: 29–34. 15027813 10.1191/0960327104ht413oa

[6] Beckman JS, Koppenol WH (1996) Nitric oxide, superoxide, and peroxynitrite: the good, the bad, and ugly. Am J Physiol 271: C1424–1437. 8944624 10.1152/ajpcell.1996.271.5.C1424

[7] Szabo C, Day BJ, Salzman AL (1996) Evaluation of the relative contribution of nitric oxide and peroxynitrite to the suppression of mitochondrial respiration in immunostimulated macrophages using a manganese mesoporphyrin superoxide dismutase mimetic and peroxynitrite scavenger. FEBS Lett 381: 82–86. 8641445 10.1016/0014-5793(96)00087-7

[8] Estevez AG, Spear N, Manuel SM, Radi R, Henderson CE, et al (1998) Nitric oxide and superoxide contribute to motor neuron apoptosis induced by trophic factor deprivation. J Neurosci 18: 923–931. 9437014 10.1523/JNEUROSCI.18-03-00923.1998 PMC6792767

[9] Salvemini D, Riley DP, Lennon PJ, Wang ZQ, Currie MG, et al (1999) Protective effects of a superoxide dismutase mimetic and peroxynitrite decomposition catalysts in endotoxin-induced intestinal damage. Br J Pharmacol 127: 685–692. 10401559 10.1038/sj.bjp.0702604 PMC1566068

[10] Shiraishi F, Curtis LM, Truong L, Poss K, Visner GA, et al (2000) Heme oxygenase-1 gene ablation or expression modulates cisplatin-induced renal tubular apoptosis. Am J Physiol Renal Physiol 278: F726–736. 10807584 10.1152/ajprenal.2000.278.5.F726

[11] Aleksunes LM, Goedken MJ, Rockwell CE, Thomale J, Manautou JE, et al (2010) Transcriptional regulation of renal cytoprotective genes by Nrf2 and its potential use as a therapeutic target to mitigate cisplatin-induced nephrotoxicity. J Pharmacol Exp Ther 335: 2–12. 20605904 10.1124/jpet.110.170084 PMC2957774

[12] Sahin K, Tuzcu M, Gencoglu H, Dogukan A, Timurkan M, et al (2010) Epigallocatechin-3-gallate activates Nrf2/HO-1 signaling pathway in cisplatin-induced nephrotoxicity in rats. Life Sci 87: 240–245. 20619277 10.1016/j.lfs.2010.06.014

[13] Sahin K, Tuzcu M, Sahin N, Ali S, Kucuk O (2010) Nrf2/HO-1 signaling pathway may be the prime target for chemoprevention of cisplatin-induced nephrotoxicity by lycopene. Food Chem Toxicol 48: 2670–2674. 20603177 10.1016/j.fct.2010.06.038

[14] Choi BM, Lim DW, Lee JA, Gao SS, Kwon DY, et al (2008) Luteolin suppresses cisplatin-induced apoptosis in auditory cells: possible mediation through induction of heme oxygenase-1 expression. J Med Food 11: 230–236. 18598163 10.1089/jmf.2007.591

[15] Domitrovic R, Cvijanovic O, Pernjak-Pugel E, Skoda M, Mikelic L, et al (2013) Berberine exerts nephroprotective effect against cisplatin-induced kidney damage through inhibition of oxidative/nitrosative stress, inflammation, autophagy and apoptosis. Food Chem Toxicol 62C: 397–406. 10.1016/j.fct.2013.09.003 24025684

[16] Mukhopadhyay P, Pan H, Rajesh M, Batkai S, Patel V, et al (2010) CB1 cannabinoid receptors promote oxidative/nitrosative stress, inflammation and cell death in a murine nephropathy model. Br J Pharmacol 160: 657–668. 20590569 10.1111/j.1476-5381.2010.00769.x PMC2931565

[17] Mukhopadhyay P, Rajesh M, Pan H, Patel V, Mukhopadhyay B, et al (2010) Cannabinoid-2 receptor limits inflammation, oxidative/nitrosative stress, and cell death in nephropathy. Free Radic Biol Med 48: 457–467. 19969072 10.1016/j.freeradbiomed.2009.11.022 PMC2869084

[18] Pan H, Mukhopadhyay P, Rajesh M, Patel V, Mukhopadhyay B, et al (2009) Cannabidiol attenuates cisplatin-induced nephrotoxicity by decreasing oxidative/nitrosative stress, inflammation, and cell death. J Pharmacol Exp Ther 328: 708–714. 19074681 10.1124/jpet.108.147181 PMC2682269

[19] Guerrero-Beltran CE, Mukhopadhyay P, Horvath B, Rajesh M, Tapia E, et al (2012) Sulforaphane, a natural constituent of broccoli, prevents cell death and inflammation in nephropathy. J Nutr Biochem 23: 494–500. 21684138 10.1016/j.jnutbio.2011.02.004 PMC3179776

[20] Chirino YI, Sanchez-Gonzalez DJ, Martinez-Martinez CM, Cruz C, Pedraza-Chaverri J (2008) Protective effects of apocynin against cisplatin-induced oxidative stress and nephrotoxicity. Toxicology 245: 18–23. 18243469 10.1016/j.tox.2007.12.007

[21] Chirino YI, Hernandez-Pando R, Pedraza-Chaverri J (2004) Peroxynitrite decomposition catalyst ameliorates renal damage and protein nitration in cisplatin-induced nephrotoxicity in rats. BMC Pharmacol 4: 20. 15458572 10.1186/1471-2210-4-20 PMC526185

[22] Mukhopadhyay P, Horvath B, Zsengeller Z, Zielonka J, Tanchian G, et al (2012) Mitochondrial-targeted antioxidants represent a promising approach for prevention of cisplatin-induced nephropathy. Free Radic Biol Med 52: 497–506. 22120494 10.1016/j.freeradbiomed.2011.11.001 PMC3253235

[23] Mukhopadhyay P, Horvath B, Kechrid M, Tanchian G, Rajesh M, et al (2011) Poly(ADP-ribose) polymerase-1 is a key mediator of cisplatin-induced kidney inflammation and injury. Free Radic Biol Med 51: 1774–1788. 21884784 10.1016/j.freeradbiomed.2011.08.006 PMC3207278

[24] Li PK, Burdmann EA, Mehta RL (2013) Acute kidney injury: Acute kidney injury–global health alert. Nat Rev Nephrol 9: 133–135. 23399583 10.1038/nrneph.2013.20

[25] Hartmann JT, Lipp HP (2003) Toxicity of platinum compounds. Expert Opin Pharmacother 4: 889–901. 12783586 10.1517/14656566.4.6.889

[26] Boogaard PJ, Mulder GJ, Nagelkerke JF (1990) Cisplatin nephrotoxicity and platinum-metallothioneins: uptake and toxicity in proximal tubular cells from rat kidney. Contrib Nephrol 83: 208–212. 2100713 10.1159/000418800

[27] Arany I, Safirstein RL (2003) Cisplatin nephrotoxicity. Semin Nephrol 23: 460–464. 13680535 10.1016/s0270-9295(03)00089-5

[28] Brozovic A, Ambriovic-Ristov A, Osmak M (2010) The relationship between cisplatin-induced reactive oxygen species, glutathione, and BCL-2 and resistance to cisplatin. Crit Rev Toxicol 40: 347–359. 20163198 10.3109/10408441003601836

[29] Pacher P, Beckman JS, Liaudet L (2007) Nitric oxide and peroxynitrite in health and disease. Physiol Rev 87: 315–424. 17237348 10.1152/physrev.00029.2006 PMC2248324

[30] Radi R, Peluffo G, Alvarez MN, Naviliat M, Cayota A (2001) Unraveling peroxynitrite formation in biological systems. Free Radic Biol Med 30: 463–488. 11182518 10.1016/s0891-5849(00)00373-7

[31] Levrand S, Vannay-Bouchiche C, Pesse B, Pacher P, Feihl F, et al (2006) Peroxynitrite is a major trigger of cardiomyocyte apoptosis in vitro and in vivo. Free Radic Biol Med 41: 886–895. 16934671 10.1016/j.freeradbiomed.2006.04.034 PMC2228266

[32] Zingarelli B, Day BJ, Crapo JD, Salzman AL, Szabo C (1997) The potential role of peroxynitrite in the vascular contractile and cellular energetic failure in endotoxic shock. Br J Pharmacol 120: 259–267. 9117118 10.1038/sj.bjp.0700872 PMC1564360

[33] Liu D, Bao F, Prough DS, Dewitt DS (2005) Peroxynitrite generated at the level produced by spinal cord injury induces peroxidation of membrane phospholipids in normal rat cord: reduction by a metalloporphyrin. J Neurotrauma 22: 1123–1133. 16238488 10.1089/neu.2005.22.1123

[34] Batinic-Haberle I, Cuzzocrea S, Reboucas JS, Ferrer-Sueta G, Mazzon E, et al (2009) Pure MnTBAP selectively scavenges peroxynitrite over superoxide: comparison of pure and commercial MnTBAP samples to MnTE-2-PyP in two models of oxidative stress injury, an SOD-specific Escherichia coli model and carrageenan-induced pleurisy. Free Radic Biol Med 46: 192–201. 19007878 10.1016/j.freeradbiomed.2008.09.042 PMC2742324

[35] Loukili N, Rosenblatt-Velin N, Li J, Clerc S, Pacher P, et al (2011) Peroxynitrite induces HMGB1 release by cardiac cells in vitro and HMGB1 upregulation in the infarcted myocardium in vivo. Cardiovasc Res 89: 586–594. 21113057 10.1093/cvr/cvq373 PMC3028979

[36] Saba H, Batinic-Haberle I, Munusamy S, Mitchell T, Lichti C, et al (2007) Manganese porphyrin reduces renal injury and mitochondrial damage during ischemia/reperfusion. Free Radic Biol Med 42: 1571–1578. 17448904 10.1016/j.freeradbiomed.2007.02.016 PMC1924492

[37] Kang JL, Lee HS, Pack IS, Leonard S, Castranova V (2001) Iron tetrakis (n-methyl-4′-pyridyl) porphyrinato (FeTMPyP) is a potent scavenging antioxidant and an inhibitor of stimulant-induced NF-kappaB activation of raw 264.7 macrophages. J Toxicol Environ Health A 64: 291–310. 11693489 10.1080/152873901316981295

[38] Nangle MR, Cotter MA, Cameron NE (2004) Effects of the peroxynitrite decomposition catalyst, FeTMPyP, on function of corpus cavernosum from diabetic mice. Eur J Pharmacol 502: 143–148. 15464100 10.1016/j.ejphar.2004.08.033

[39] Dhar A, Kaundal RK, Sharma SS (2006) Neuroprotective effects of FeTMPyP: a peroxynitrite decomposition catalyst in global cerebral ischemia model in gerbils. Pharmacol Res 54: 311–316. 16877004 10.1016/j.phrs.2006.06.009

[40] Baliga R, Zhang Z, Baliga M, Ueda N, Shah SV (1998) In vitro and in vivo evidence suggesting a role for iron in cisplatin-induced nephrotoxicity. Kidney Int 53: 394–401. 9461098 10.1046/j.1523-1755.1998.00767.x

[41] Zielonka J, Sikora A, Joseph J, Kalyanaraman B (2010) Peroxynitrite is the major species formed from different flux ratios of co-generated nitric oxide and superoxide: direct reaction with boronate-based fluorescent probe. J Biol Chem 285: 14210–14216. 20194496 10.1074/jbc.M110.110080 PMC2863194

[42] Beckman JS, Beckman TW, Chen J, Marshall PA, Freeman BA (1990) Apparent hydroxyl radical production by peroxynitrite: implications for endothelial injury from nitric oxide and superoxide. Proc Natl Acad Sci U S A 87: 1620–1624. 2154753 10.1073/pnas.87.4.1620 PMC53527

[43] Radi R, Beckman JS, Bush KM, Freeman BA (1991) Peroxynitrite-induced membrane lipid peroxidation: the cytotoxic potential of superoxide and nitric oxide. Arch Biochem Biophys 288: 481–487. 1654835 10.1016/0003-9861(91)90224-7

[44] Radi R, Beckman JS, Bush KM, Freeman BA (1991) Peroxynitrite oxidation of sulfhydryls. The cytotoxic potential of superoxide and nitric oxide. J Biol Chem 266: 4244–4250. 1847917

[45] Crow JP, Beckman JS (1995) The role of peroxynitrite in nitric oxide-mediated toxicity. Curr Top Microbiol Immunol 196: 57–73. 7634825 10.1007/978-3-642-79130-7_7

[46] Cassina AM, Hodara R, Souza JM, Thomson L, Castro L, et al (2000) Cytochrome c nitration by peroxynitrite. J Biol Chem 275: 21409–21415. 10770952 10.1074/jbc.M909978199

[47] Hortelano S, Alvarez AM, Bosca L (1999) Nitric oxide induces tyrosine nitration and release of cytochrome c preceding an increase of mitochondrial transmembrane potential in macrophages. FASEB J 13: 2311–2317. 10593878 10.1096/fasebj.13.15.2311

[48] Aulak KS, Miyagi M, Yan L, West KA, Massillon D, et al (2001) Proteomic method identifies proteins nitrated in vivo during inflammatory challenge. Proc Natl Acad Sci U S A 98: 12056–12061. 11593016 10.1073/pnas.221269198 PMC59826

[49] Yamakura F, Taka H, Fujimura T, Murayama K (1998) Inactivation of human manganese-superoxide dismutase by peroxynitrite is caused by exclusive nitration of tyrosine 34 to 3-nitrotyrosine. J Biol Chem 273: 14085–14089. 9603906 10.1074/jbc.273.23.14085

[50] Trujillo M, Radi R (2002) Peroxynitrite reaction with the reduced and the oxidized forms of lipoic acid: new insights into the reaction of peroxynitrite with thiols. Arch Biochem Biophys 397: 91–98. 11747314 10.1006/abbi.2001.2619

[51] Gozzelino R, Jeney V, Soares MP (2010) Mechanisms of cell protection by heme oxygenase-1. Annu Rev Pharmacol Toxicol 50: 323–354. 20055707 10.1146/annurev.pharmtox.010909.105600

[52] Keyse SM, Tyrrell RM (1989) Heme oxygenase is the major 32-kDa stress protein induced in human skin fibroblasts by UVA radiation, hydrogen peroxide, and sodium arsenite. Proc Natl Acad Sci U S A 86: 99–103. 2911585 10.1073/pnas.86.1.99 PMC286411

[53] Applegate LA, Luscher P, Tyrrell RM (1991) Induction of heme oxygenase: a general response to oxidant stress in cultured mammalian cells. Cancer Res 51: 974–978. 1988141

[54] Ryter SW, Alam J, Choi AM (2006) Heme oxygenase-1/carbon monoxide: from basic science to therapeutic applications. Physiol Rev 86: 583–650. 16601269 10.1152/physrev.00011.2005

[55] Domitrovic R, Cvijanovic O, Pugel EP, Zagorac GB, Mahmutefendic H, et al.. (2013) Luteolin ameliorates cisplatin-induced nephrotoxicity in mice through inhibition of platinum accumulation, inflammation and apoptosis in the kidney. Toxicology. 10.1016/j.tox.2013.05.015 23770416

[56] Kim MG, Yang HN, Kim HW, Jo SK, Cho WY, et al (2010) IL-10 mediates rosiglitazone-induced kidney protection in cisplatin nephrotoxicity. J Korean Med Sci 25: 557–563. 20357998 10.3346/jkms.2010.25.4.557 PMC2844593

[57] Ozkaya O, Yavuz O, Can B, Dilek M, Savli E, et al (2010) Effect of rosiglitazone on cisplatin-induced nephrotoxicity. Ren Fail 32: 368–371. 20370454 10.3109/08860221003611729

[58] Lee S, Kim W, Moon SO, Sung MJ, Kim DH, et al (2006) Rosiglitazone ameliorates cisplatin-induced renal injury in mice. Nephrol Dial Transplant 21: 2096–2105. 16728429 10.1093/ndt/gfl194

[59] Kumar P, Prashanth KS, Gaikwad AB, Vij M, Barua CC, et al (2013) Disparity in actions of rosiglitazone against cisplatin-induced nephrotoxicity in female Sprague-Dawley rats. Environ Toxicol Pharmacol 36: 883–890. 24001946 10.1016/j.etap.2013.08.004

[60] Broeders N, Abramowicz D (2002) Peroxisome proliferator-activated receptors (PPARs): novel therapeutic targets in renal disease. Kidney Int 61: 354–355. 11786122 10.1046/j.1523-1755.2002.00129.x

[61] Mukhopadhyay P, Rajesh M, Batkai S, Kashiwaya Y, Hasko G, et al (2009) Role of superoxide, nitric oxide, and peroxynitrite in doxorubicin-induced cell death in vivo and in vitro. Am J Physiol Heart Circ Physiol 296: H1466–1483. 19286953 10.1152/ajpheart.00795.2008 PMC2685360

[62] Liaudet L, Rosenblatt-Velin N, Pacher P (2013) Role of peroxynitrite in the cardiovascular dysfunction of septic shock. Curr Vasc Pharmacol 11: 196–207. 23506498

[63] Pacher P, Szabo C (2006) Role of peroxynitrite in the pathogenesis of cardiovascular complications of diabetes. Curr Opin Pharmacol 6: 136–141. 16483848 10.1016/j.coph.2006.01.001 PMC2228269

[64] Pacher P, Schulz R, Liaudet L, Szabo C (2005) Nitrosative stress and pharmacological modulation of heart failure. Trends Pharmacol Sci 26: 302–310. 15925705 10.1016/j.tips.2005.04.003 PMC2228264

[65] Pacher P, Obrosova IG, Mabley JG, Szabo C (2005) Role of nitrosative stress and peroxynitrite in the pathogenesis of diabetic complications. Emerging new therapeutical strategies. Curr Med Chem 12: 267–275. 15723618 10.2174/0929867053363207 PMC2225483

