# Supplementary figures and images for: Protective Effect of Metalloporphyrins against Cisplatin-Induced Kidney Injury in Mice
Source: PLoS One. 2014 Jan 14;9(1):e86057. doi: 10.1371/journal.pone.0086057 (PMC3891880; doi:10.1371/journal.pone.0086057)

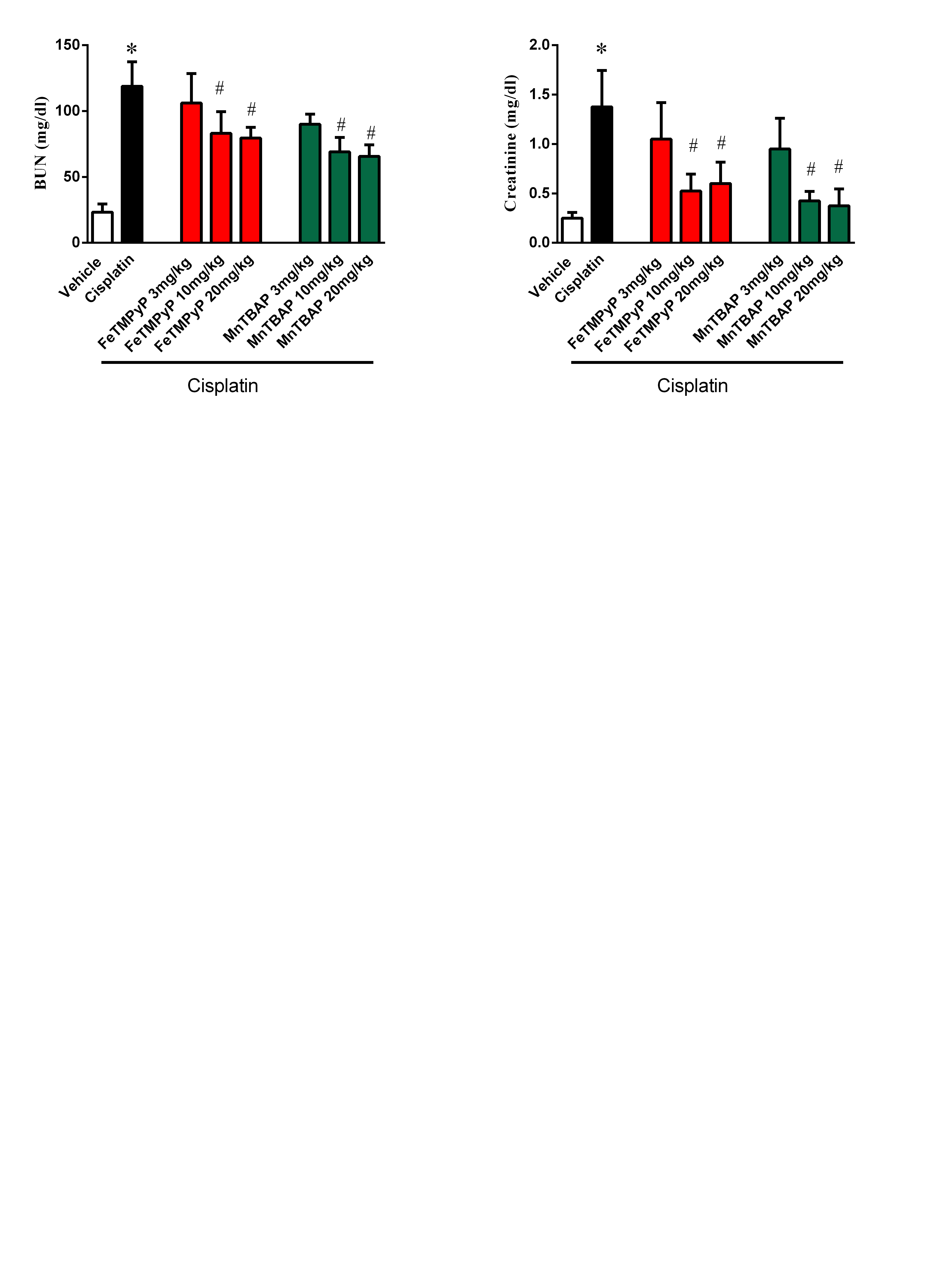

Supplement: Figure S1 — Dose Dependent effect of Metalloporphyrins on Cisplatin-Induced Renal Dysfunction in Mice. Cisplatin-induced significant renal dysfunction measured by the levels of BUN and creatinine. BUN and Creatinine were measured in serum from mice at 72 h after cisplatin administration. Cisplatin administration resulted in severe kidney injury which was attenuated by FeTMPyP and MnTBAP treatment in a dose dependent manner. Results are mean ± S.E.M. n = 4–5/group. *p<0.05 versus vehicle; and #p<0.05 versus cisplatin. (TIF) [file pone.0086057.s001.tif]
